# Noxa and Puma genes regulated by hTERT promoter can mitigate growth and induce apoptosis in hepatocellular carcinoma mouse model

**DOI:** 10.7150/jca.70282

**Published:** 2022-03-28

**Authors:** Tongjian Zhao, Chang Zhao, Yuanhua Lu, Jian Lin, Yafei Tian, Yongjun Ma, Jialin Li, Hugang Zhang, Weiqun Yan, Ping Jiao, Jie Ma

**Affiliations:** Department of Regenerative Medicine, School of Pharmaceutical Sciences, Jilin University, Changchun 130021, China.

**Keywords:** *Noxa*, * Puma*, telomerase reverse transcriptase promoter, liver cancer, gene therapy

## Abstract

With significant high incidence and death rates, liver cancer has become one of the most common cancers all over the world. Hence, novel strategies are needed for the management of this malignancy. Apoptotic related proteins *Noxa* and *Puma* are the members of BH3-only family. In this study, human *Noxa* or *Puma* coding sequences have been inserted into plasmid pcDNA 3.1 regulated by human TERT promoter. The transfection of HepG2 cells with pcTERT-Noxa or pcTET-Puma resulted in the significant suppression of cell proliferation as well as finally led to apoptosis via mitochondrial and death receptor pathways, and also exhibited significantly reduced the ability of invasion and metastasis. Moreover, an *in vivo* study revealed that intratumoral injections of pcTERT-Noxa or pcTERT-Puma plasmids effectively suppressed the tumor growth and can exhibit anti-neoplastic effects by recruiting *CD3*, *CD8*, *CD45* positive T lymphocytes in the tumor tissues. Overall, our findings illustrated that pcTERT-Noxa and pcTERT-Puma may exhibit significant anti-tumor effects both *in vivo* and *in vivo*.

## Introduction

Liver cancer is a malignant tumor with the sixth highest incidence rates and the third leading cause of cancer death in 2020 all over the world, of which about 906,000 new cases were diagnosed and 830,000 deaths were reported 1. Although surgical techniques combined with radiotherapy and chemotherapy have become more sophisticated, the incidence and mortality have remained largely unchanged for the liver cancer due to the poor prognosis 2, 3. Gene targeted therapies can enable the transfer of therapeutic molecules into cells to modify the expression of specific molecular targets, and thus may serve as promising strategy for cancer treatment 4. However, finding novel therapeutic targets can be quite difficult for liver cancer. Thus, the early detection and the discovery of new targets are urgently needed to reduce the damage inflicted by this mortality.

Telomerase belongs to the reverse transcriptase family which contains 5'TTAGGG3' repeats that mediates proper maintenance of telomere in nearly 90% of human carcinomas 5. Telomerase is a complex enzyme containing an internal non-coding RNA component and proteins capable of activating telomere-specific reverse transcriptase 6. Most normal human cells are unable to maintain immortality because of the transcriptional suppression of the rate-limiting step by relatively lower expression of processivity factor and the lack of catalytic component in telomerase reverse transcriptase (*hTERT*) gene 7. A large number of studies have been carried out to target telomerase for development of effective anti-cancer 8-10. A sequence consisting of 181 bp in upstream region of the transcriptional starting point of hTERT is the core promoter sequence. This promoter sequence also contains the domain that can interact with multiple transcription factors, such as *E box* (CACGTG) binding factor and *SP1* (specificity protein 1) 11.

Apoptosis is a programmed cell death process that can be effectively triggered by stress, DNA damage and toxic stimuli 12. This process involves the release of cytochrome *c* from the mitochondria and finally recruitment of the *cleaved Caspase-3* as an effector 13, which is regulated by a balance in the activity of the pro-apoptotic and anti-apoptotic proteins of the *BCL-2* family 14. The proteins belonging to the B-cell lymphoma-2 (*Bcl-2*) family have been reported to alleviate the apoptosis of cells by distinct mechanism(s) 15. *Bcl-2* family consists of various pro-apoptotic proteins and anti-apoptotic proteins. The pro-apoptotic proteins of *Bcl-2* family contain BH 1-3 conserved domains or *BH3* domain-only. Recently, a number of studies have investigated the role of BH3-only proteins in regulating apoptosis through the mitochondrial pathway 16-18, especially for the treatment of different cancers (16). The BH3-only proteins family can lead to apoptosis by altering the levels of the pro-survival *BCL-2* proteins family 19. For example, it has been reported that the apoptosis of human glioblastoma by type I interferons is mediated through the upregulation of BH3-only proteins-NOXA, which can thereby alter the expression of *BCL-2* proteins family member *MCl-1* (Myeloid Cell Leukemia-1) 20. Moreover, BH3-only proteins can eliminate cancer cells as increased Puma expression by *TGF-β* (transforming growth factor-β) stimulation has been reported to lead to a rapid cell death of Burkitt's lymphoma cells 21. However, far too little attention has been paid in investigating that whether *Noxa* or* Puma* could kill cancer cells specifically.

Gene therapy is currently considered as a promising treatment for multiply diseases after nearly 50 years when the concept was first proposed. Due to the advantages in manipulating gene expression, gene therapy has come up as an appealing way to achieve the delivery of therapeutic genes into cancer cells 22. Currently, some new approaches emerged with gene promoters have been taken into considered for tumoral gene treatment due to abundant transcriptional activity in cancer cells, but low or absence of activity in the normal cells 23, 24. Human telomerase reverse transcriptase (hTERT) promoter can be repressed in most human somatic normal cells, which consequently results in telomerase silencing but has been primarily detected in the malignant cells 25. Therefore, hTERT promoter is a relative optimum choice for tumor gene therapy because of its specific tumor-targeting characteristic. Thus, we describe the generation of pcDNA3.1 harboring *Noxa/Puma* genes under the control of hTERT promoter that can effectively targeting hepatoma cells HepG2 and tumor growth in a preclinical model. These finding showed that substantial alterations in the expression of *Noxa* or *Puma* without any other treatments can also exhibit significant anti-tumor effects and thus offers new insights that, could be relevant to the field of liver cancer drug development.

## Materials and Methods

### Reagents

The HepG2 and HL-7702 cell lines were obtained from American Type Tissue Culture Collection (Manassas, VA, USA). H22 hepatoma cells were provided by the Shanghai Institute of Materia Medica, Chinese Academy of Sciences (Shanghai, China). Fetal bovine serum (FBS) was purchased from Gibco (Waltham, MA, USA). DMEM (Dulbecco's modified Eagle medium) was obtained from the Hyclone (Logan, UT, USA). Opti-MEM (Opti-minimal essential medium) reduced serum, Lipofectamine 2000 and Trizol reagent were purchased from Invitrogen Life Technology (Carlsbad, CA, USA). Protease inhibitor cocktail tablet, Annexin-V-FLUOS staining kit was procured from Roche Diagnostics (Indianapolis, IN, USA) and FastStart Universal SYBR Green Master, Hochest 33258 was obtained from Beyotime Institute of Biotechnology (Shanghai, China). PrimeScript TM RT reagent kit with gDNA Eraser and Premix Ex TaqTM PCR kits (Real-Time) were purchased from TaKaRa (Dalian, Liaoning, China). Enhanced chemiluminesence reagent was obtained from Pierce Biotechnology (Rockford, IL, USA). As for quantification for total protein in cytoplasm, Pierce BCA protein assay kit was used and was purchased from Thermo Scientific (Waltham, MA, USA). Antibodies against *Bcl-2*, *Bax*,* cleaved Caspase-9* and *cleaved Caspase3* were purchased from Cell Signaling Technology (Beverly, MA, USA). *Caspase-8* (Cleaved-Asp384) and *PARP1* antibodies were obtained from Affinity Biosciences (Cincinnati, OH, USA). Antibodies against *CD3*, *CD8*, *CD45*, *GAPDH* and horse radish peroxidase (HRP)-labelled conjugated secondary antibodies to rabbit, mouse and goat primary antibody were purchased from Santa Cruz Biotechnology (Danvers, MA, USA). CellTiter96^®^ AQueous One Solution Cell Proliferation Assay kit was obtained from Promega (Madison, WI, USA).

### Construction of pcTERT-Noxa, pcTERT-Puma vector

The recombinant plasmids encoding *Noxa* or *Puma* were constructed using a pcDNA3.1+ plasmid containing the hTERT promoter that has already been previously constructed in our laboratory 26. *Noxa* or *Puma* coding genes were synthesized by GeneWIZ Biotech Co., Ltd (Suzhou, China). *Noxa* with double enzyme cutting sites MluI and XbaI or *Puma* with double enzyme cutting sites HindIII and XbaI were inserted into the corresponding enzyme linearized pcDNA3.1+ plasmid to regenerate recombinant plasmids named as pcTERT-Noxa or pcTERT-Puma. The recombinant plasmid contained no additional genes except for enhancer and hTERT promoter that was named as pcTERT.

### Cell culture and treatments

The HepG2 and HL7702 cells were cultured in DMEM with 10% FBS at 37°C under 5% CO_2_ condition. After the cells had grown approximately to 80% confluence in 10 cm dish, they were collected and seeded into 6-well plates at a density of 5×10^5^ cells/well. The medium was changed into opti-MEM reduced serum at least 2h before the transfection. The HepG2 and HL7702 cells were transfected with pcTERT-Noxa, pcTERT-Puma or pcTERT plasmids respectively using Lipofectamine 2000 as the manufacturer's guidelines and incubated for 24 h, 48 h and 72 h before being analyzed for the various assays.

### Cell proliferation assay

The cell viability was determined via the 3-(4,5-dimethylthiazol-2-yl)-5-(3-carboxymethoxyphenyl)-2-(4-sulfophenyl)-2H-tetrazolium, inner salt (MTS) assay using a CellTiter96^®^ Aqueous assay proliferation kit. Briefly, HepG2 and HL7702 cells were plated in 96-well plates at a density of 4×10^3^ cells/well and cultured for 12h. Thereafter the cells were transfected with pcTERT-Noxa, pcTERT-Puma or pcTERT plasmids and incubated for 24 h, 48 h and 72 h. After that, 20 μl MTS solution was added in fresh 100μl medium after replacing the original medium and the plate was incubated for another 2 h at 37℃ under 5% CO_2_. The microplate reader (Bio-Tek, Winooski, VT, USA) was then used to measure the absorbance of each well at 490 nm.

### Hoechst 33258 staining assay

The cells were transfected with pcTERT, pcTERT-Noxa or pcTERT-Puma plasmids for 24 h and then harvested, and fixed with 4% paraformaldehyde for 30 min at 25°C. After washing three times with PBS, the cells were stained with Hoechst 33258 for 10 min followed by additional washing with PBS for three times. Finally, the stained nuclei were visualized under a fluorescence microscope (Olympus, Tokyo, Japan).

### Annexin-V-FLUOS/propidium iodide (PI) analysis

HepG2 and HL7702 cells transfected with pcTERT, pcTERT-Noxa, pcTERT-Puma plasmids for 72 h and then were washed with PBS thrice and then the cells were collected both from the plates and the supernatant. Thereafter the labeling solution of Annexin-V-FLOUS was added and the cells were incubated for 30 min at 37℃. The population of apoptotic cells was determined by flow cytometry and analyzed by CellQuest software (BD, FranklinLaked, NJ, USA).

### RNA preparation and quantitative real-time PCR

Total RNA was extracted by Trizol reagent and oligo primers were used for reverse transcription based on the recommended protocol. The Real-time PCR was carried out using SYBR Green Master on StepOnePlus system (Applied Biosystems, Carlsbad, CA, USA). The primer sequences used were synthesized by Sangon Biotech (Shanghai, China) and have been described in Table 1. The relative mRNA expression was normalized by comparison with *GAPDH* and calculated with the 2^-ΔΔCt^ method.

### Western blot analysis

HepG2, and HL7702 cells or the tumor tissues obtained from H22-bearing mice transfected with pcTERT, pcERT-Noxa or pcTER-Puma plasmids were lysed with cold lysis buffer containing a protease inhibitor cocktail. BCA (bicinchoninic acid) protein assay kit was used to measure the concentration of the protein in the lysates. Then, 50 μg whole-cell proteins were separated by 12% sodium dodecyl sulfate polyacrylamide (SDS-PAGE) gels followed by transfer onto a PVDF (polyvinylidene fluoride) membrane. After that, the membranes were blocked with 2% BSA (bovine serum albumin) and incubated with corresponding primary antibodies overnight at 4 ℃ upon a shaker and thereafter incubated with the corresponding secondary antibody at appropriate concentration with 1:20000 at 25 ℃ for 1 h. The protein bands were detected by enhanced chemiluminescence (ECL) reagent on Tanon 5200 Multi fluorochrome image system (Tanon, Shanghai, China) and quantified using Image J software.

### Clonogenic assay

HepG2 cells were collected after the transfection with pcTERT, pcTERT-Puma or pcTERT-Noxa plasmids for 72 h and then seeded into 6-well plate at 100 cells/ well and cultured for another 3 weeks until single cell colonies were observed. For this assay, each treatment group was seeded in the triplicate wells. Consequently, HepG2 cells were washed twice with PBS and fixed with 4 % paraformaldehyde for additional 15 min. Thereafter, the cells were stained with 0.05 % crystal violet for another 15min. A colony was considered as viable if more than 50 cells were observed. The efficiency was calculated as the number of staining colonies divided by the number of seeded cells using the following formula:

Cloning forming efficiency (%) = (Number of colonies formed/the number of cells that were seeded) ×100 %

### Scratch assay

To investigate the wound healing effect of pcTERT, pcTERT-Puma, or pcTERT-Noxa plasmids in HepG2 cells, the cells transfected with the above-indicated plasmids were plated at a density of 7×10^4^ cells in 24-well plates. When the HepG2 cells grew to around 80% confluence, they were scratched by using pipet and washed with PBS to remove the cell debris and cultured for another 72 h. The cell mobility was monitored with a microscope after every 24 h. The relative cell mobility at each time point was analyzed using Image J software in experimental triplicates of three independent experiments.

Cell mobility rate (%) = (the area of scratch at each time point/primary scratch area) ×100%

### Invasion assay using transwell chambers

The HepG2 cells were transfected with above-mentioned plasmids for 72 h after which they were harvested after trypsinization. 1.5 × 10^5^ cells transfected with pcTERT, pcTERT-Puma or pcTERT-Noxa plasmids for 72h were plated in 200 μl serum-free medium on the top chamber of 12-well plate with the matrigel basement membrane (New York, USA) for invasion assay or without for migration assay while 600 μl DMEM with 10% FBS was added in the lower chamber. After incubation for 24 h, the cells were removed from the top chamber. The chambers were washed with PBS thrice and the cells on the bottom were collected and fixed with 4% paraformaldehyde for 30 min. The cells were then stained with 0.05% crystal violet solution for another 15 min. The HepG2 cells in the lower chamber were then counted under the microscope (Olympus IX83, Tokyo, Japan). At last, the cells stained with 0.05% crystal violet were dissolved in 30% acetate and absorbance at OD570 was measured for each group.

### Mice and Tumor models

Six to eight week old male BALB/c mice (HUAFUKANG Bioscience, Beijing, China) were housed 5 per cage under a 12 h-cycle of light/dark with water and food accessible. H22 cells (3×10^5^/mouse) were injected into left subaxillary subcutaneously and the tumor model was established after 10 days. 100 μl lipofectamine 2000 harboring 50 μg plasmid of pcTERT, pcTERT-Noxa or pcTERT-Puma was intratumorally injected into the BALB/c mice three times with each interval of 72 h after the mice has developed a xenograft tumor after 10 days. All the mice were sacrificed in 3 days after receiving the last intratumoral plasmid injection. The tumor volume was calculated according to the formula (cm^3^) = length×width^2^/2. The mouse tumor tissues were isolated and stored for western blot analysis, immunohistochemistry and HE staining at a later time. All the animal experiments were approved by the Animal Experiment Ethics Committee of Jilin University.

### Immunohistochemistry (IHC)

Immunohistochemical staining was performed on formalin-fixed paraffin-embedded tissue sections, which had been cut into 4 μm sections. Firstly, the slides were deparaffinized with dimethylbenzene, followed by hydration and exposure to gradient ethanol. Thereafter 3% H_2_O_2_ was used to eliminate peroxidase and the slides were boiled for 15 min followed by treatment with 0.01M sodium citrate to retrieve antigen. After 30 min blocking with 5% BSA, the primary antibodies (*CD3*, *CD8*, and *CD45*) were applied at a ratio of 1:100 and incubated overnight at 4°C in a humidity chamber. After that, the slides were washed several times with PBS and incubated with corresponding secondary antibody for 1h. Thereafter, the slides were developed with DAB (diaminobenzidine), stained with hematoxylin and cleaned in the flowing water. Finally, the slides were examined with microscope to determine the expression of various proteins.

### H&E (hematoxylin-eosin) staining

The 5 μm -thick paraffin embedded sections fixed in 4% paraformaldehyde were cut and stained with H&E according to the conventional procedures 27.

### Evaluation of immunostaining results

The intensity of IHC was scored by three sophisticated pathologists with no prior knowledge of the details in the mice tumors. Three representative areas of each group were microscopically observed (magnification, ×100) and numbered each positively-stained cells. Cytoplasmic staining was considered as positive according to the corresponding primary antibody instruction. The expression level of CD3, CD8 and CD45 in each slide was semi quantitatively scored on both staining intensity and the percentage of stained cells. The staining intensity was developed on the following criteria: None (score: 0), mild (score:1), moderate (score: 2) and strong (score: 3). The percentage of positive cells was scored as follows: <5% (score: 0), 6-25% (score:1), 26-50% (score: 2), 51-75% (score: 3) and >75% (score: 4). The immunoreactivity scores (IRS) were calculated by adding the immunostaining percentages and immunostaining intensity scores together 28.

### Statistical Analysis

All the data was collected as means ± standard error (SE). Statistical analyses were performed using the two-sample Student's t tests and ANOVAs followed by an LSD post hoc test. *P* < 0.05 was considered as the threshold of statistical significance.

## Results

### Overexpression of Noxa or Puma induced morphological changes in HepG2 cells

**Figure 1:**
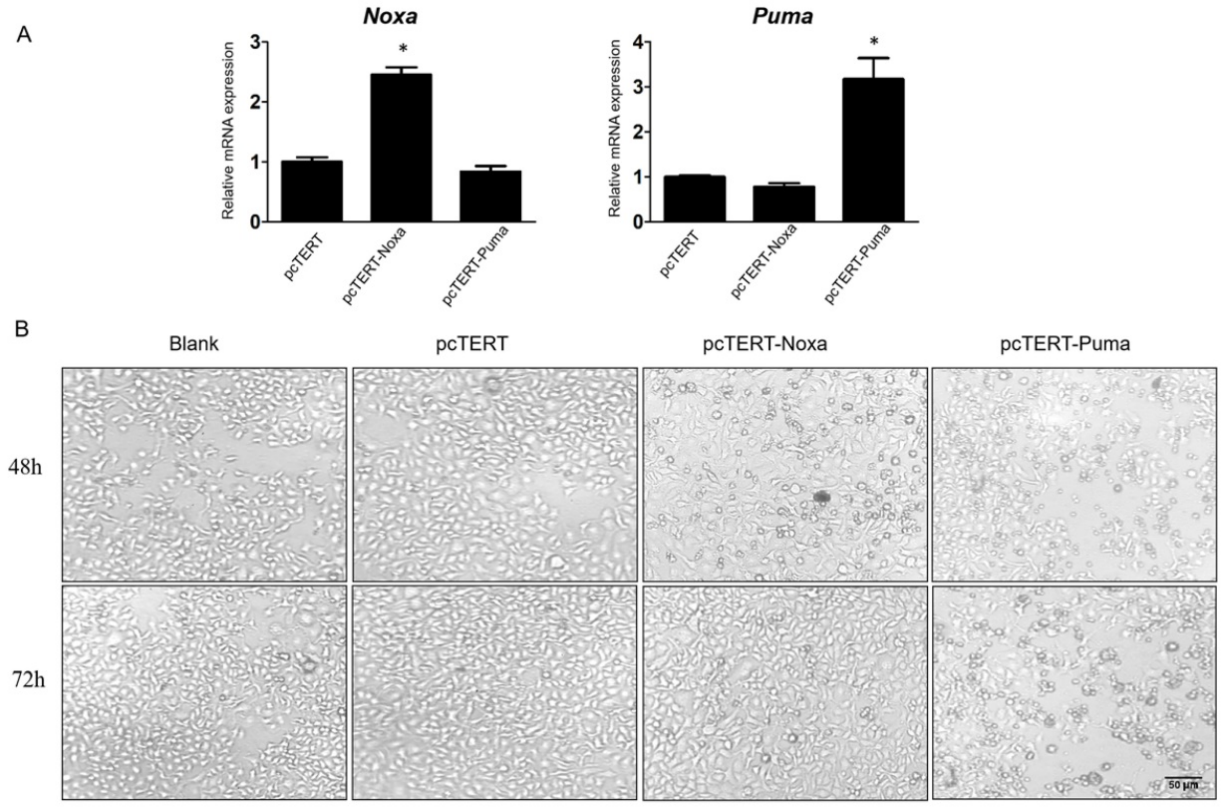
Morphological changes in HepG2 cells caused by the overexpression of *Noxa* or *Puma* A. Realtime PCR analysis the Noxa or Puma expression in HepG2 cells transfected with pcTERT, pcTERT-Noxa or pcTERT-Puma for 72h. B. HepG2 cells with pcTET-Noxa or pcTERT-Puma 48h- transfection displayed significant morphological changes that became more pronounced after 72 h (original magnification, ×100) Data are showed as the mean ± SE, * *P* < 0.05, pcTERT-Noxa or pcTERT-Puma group *vs.* pcTERT group. The experiments were repeated three times with similar results and the result of one representative experiment has been shown.

In order to confirm the possible functions of *Noxa* and *Puma*, pcDNA3.1 plasmid with hTERT promoter harboring *Noxa* or *Puma* genes were expressed in HepG2 cells while pcTERT acted potentially as a control plasmid. HepG2 cells were transiently transfected with hTERT, hTERT-Noxa or hTERT-Puma respectively for 72 h. As depicted in Fig 1A, the relative mRNA expression of *Noxa* or *Puma* was obviously increased by at least 2-fold as compared with pcTERT controls. Apoptosis is a form of cell death accompanied with significant morphological changes, thus the optical microscopy has been extensively used for distinguishing apoptotic and normal cells 29. Consistent with the results, it was found that the pcTERT-Noxa or pcTERT-Puma plasmid triggered substantial morphological changes like cell shrinkage, rounded appearance, as well as nuclear condensation 48 h after transfection and these changes become more pronounced post 72 h as shown in Fig 1B. Conversely, the HepG2 cells transfected with control pcTERT showed normal morphology consisting of regular shape, uniform border and tight intercellular junction. These results suggested that *Puma* and *Noxa* could lead to substantial apoptotic morphological variations in HepG2 cells in a time-dependent manner.

### Noxa or Puma can lead to apoptosis specifically in HepG2 cells

**Figure 2:**
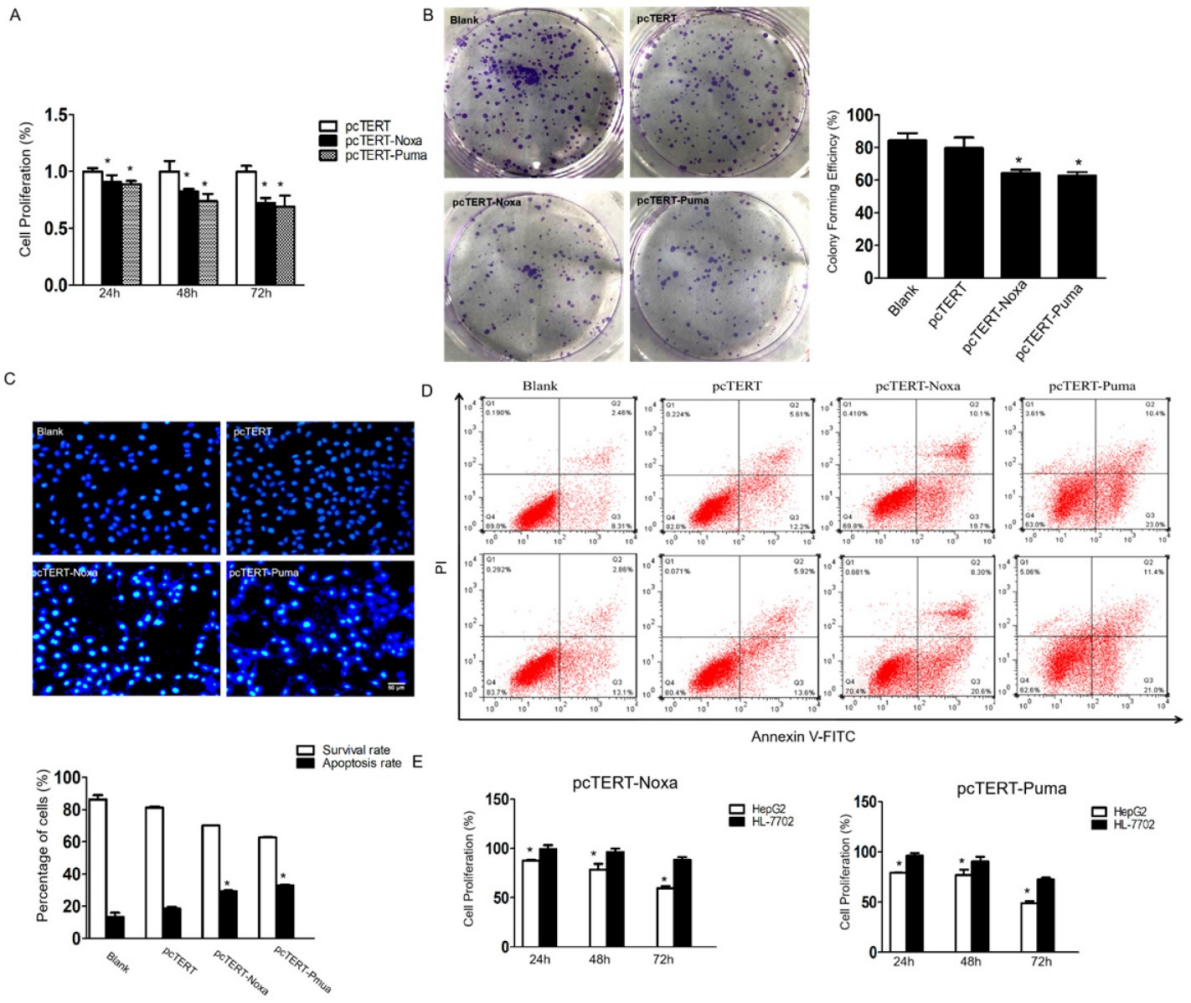
pcTERT-Noxa or pcTERT-Puma promote apoptosis of HepG2 cells (A) The cell proliferation rate of HepG2 cells transfected with pcTERT-Noxa or pcTERT-Puma for 24, 48 and 72 h were analyzed by MTS assay and (B) by colony formation assay 72 h post transfection. (C) Apoptosis in HepG2 cells 72 h after transfection with pcTERT-Noxa or pcTERT-Puma was measured by Hoechst 33258 staining (D) and quantified via Annexin V and propidium iodide (PI) staining method. (E) The differences in survival rate between HepG2 and HL-7702 cells 24, 48 and 72h after transfection of pcTERT-Noxa or pcTERT-Puma was assessed by MTS assay. * *P* < 0.05, pcTERT-Noxa or pcTERT-Puma group *vs.* pcTERT group; HepG2 cells *vs.* HL-7702 cells**.** All experiments were repeated three times with similar results and the result of one representative figure has been presented.

The MTS assay was performed to determine whether *Noxa* or *Puma* could modulate the growth of HepG2 cells. As shown in Fig 2A, the proliferation of HepG2 cells that have already transfected with pcTERT-Noxa or pcTERT-Puma was significantly decreased to (90.71±0.06) % and (88.72±0.03) % as compared to those transfected with pcTERT control for 24 h. The viability further declined to (82.36±0.02) % and (73.74±0.07) % for 48 h and reached to the minimal level at (72.31±0.04) % and (69.05±0.09) % after 72h-treatment, thus displaying a time-dependent response. The colony formation assay is a fundamental method to evaluate *in vitro* cell growth ability 30. We next analyzed colony formation ability of HepG2 cells, which can also indicate the changes in the pattern of cell growth. The results demonstrated that the effect of colony forming efficiency of HepG2 cells upon transfection with pcTERT-Noxa (64.33±2.08) % or pcTERT-Puma (62.67±2.31) % were obviously reduced as compared with cells only transfected with pcTERT (79.67±6.43) % or non-vector (84.33±4.51) % (Fig 2B). Consistently, in Hoechst staining, few cells in blank or pcTERT-treated group displayed disruption of nucleus. Nevertheless, majority of cells in pcTERT-Noxa or pcTERT-Puma-treated group were observed to be densely stained at 72 h, which clearly indicated the disruption of nucleus (Fig 2C). Moreover, to further explore the apoptosis of HepG2 cells transfected with pcTERT-Noxa or pcTERT-Puma, apoptotic cells were quantified by Annexin-V-FLOUS using a flow cytometer. As shown in Fig 2D, pcTERT-Noxa or pcTERT-Puma group obviously showed a significant increase in the apoptosis rate to (29.35±0.64) % and (32.9±0.71) % against pcTERT treated group (18.67±1.21) %, thereby suggesting that Noxa or Puma harboring by hTERT promoter could stimulate substantial apoptosis in HepG2 cells. For further exploring the specific targeting effect of pcTERT-Noxa or pcTERT-Puma towards cancer cells, normal liver cells HL-7702 were used as a control and the same transfection procedure as mentioned above by using pcTERT-Noxa or pcTERT-Puma for 24 h, 48 h and 72 h was repeated. It was found that HL-7702 cells upon treatment of pcTERT-Noxa or pcTERT-Puma exhibited a minimal change in the proliferation with (99.55±3.71) % and (96.01±2.96) % in 24h, (96.34±3.60) % and (90.51±4.48) % in 48h, (88.56±2.62) % and (72.70±1.90) % in 72h (Fig 2E), which clearly illustrated that pcTERT-Noxa or pcTERT-Puma can induce apoptosis specifically in HepG2 cells, with little effect on the survival of normal liver HL-7702 cells.

### pcTERT-Noxa or pcTERT-Puma inhibited migration and invasive potential of HepG2 cells

**Figure 3:**
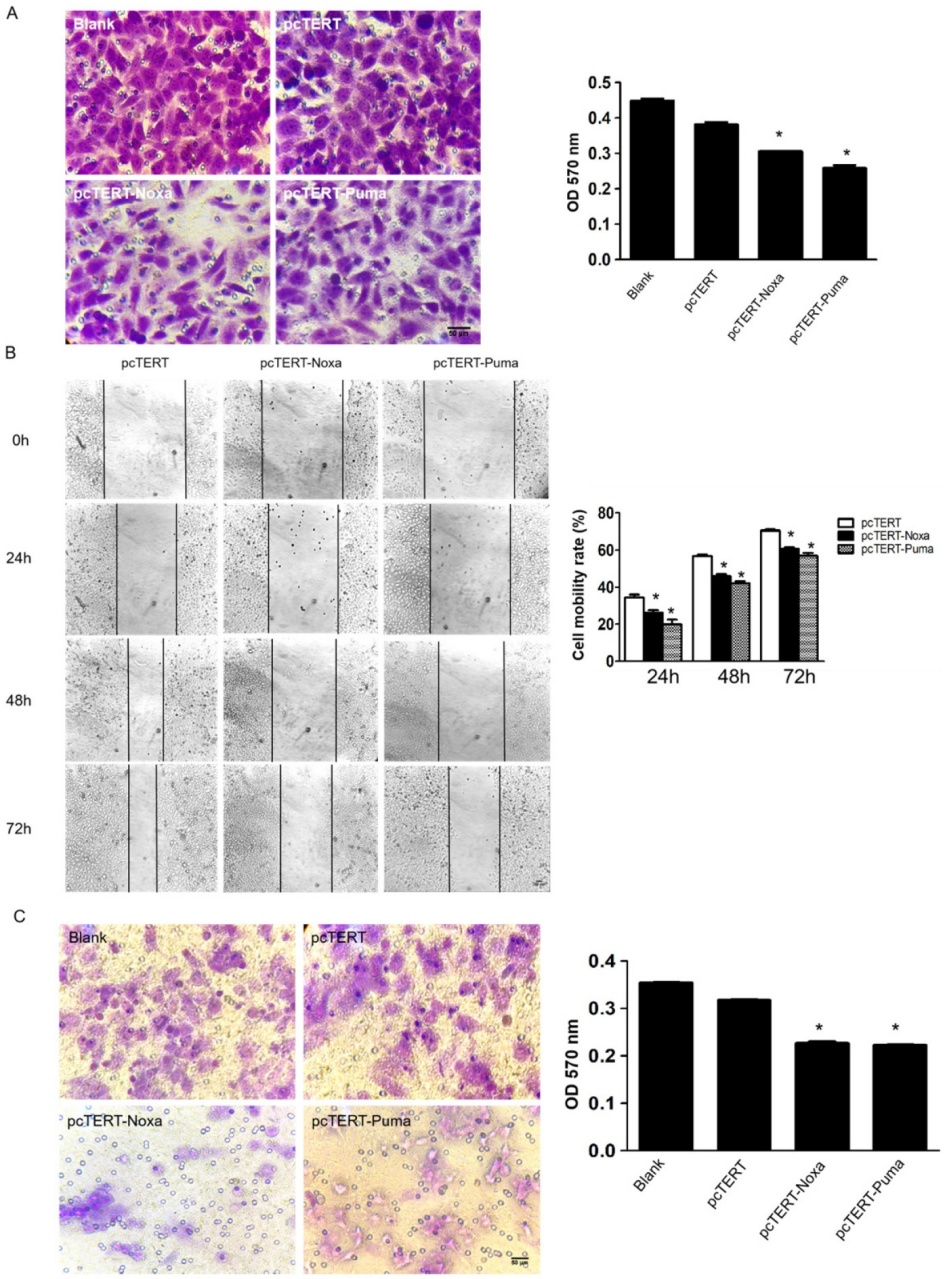
pcTERT-Noxa and pcTERT-Puma inhibited migration and invasiveness on HepG2 cells (A) The potential of migration was determined by transwell migration assay 72 h post-transfection with pcTERT-Noxa or pcTERT-Puma. The cells attached to the interior surface of the membrane of each group were stained with 0.05% crystal violet and photographed under the microscope (100×) (left) and motility rates were calculated by microplate reader at 570 nm absorbance after the crystal violet dye was dissolved by 30% acetic acid (right). (B) The scratch healing images (100×) of HepG2 cells transfected with pcTERT-Noxa or pcTERT-Puma for 0, 24, 48 and 72h were taken under the optical microscope (left) and analyzed for cell mobility rate in each group (right). (C) HepG2 cells that stimulated following 72h pcTERT-Noxa or pcTERT-Puma treatment were found to invade through the membranes, and then the cells were stained with 0.05% crystal violet (100×) (left). The rate of invasion was detected by measuring absorbance at 570 nm using a microplate reader after dissolving crystal violet-stained cells in 30% acetic acid (right). * *P*<0.05, pcTERT-Noxa *vs.* pcTERT; pcTERT-Puma *vs.* pcTERT**.** All pictures were captured three times with similar results to obtain error bars and one representative picture of each group has been shown.

**Figure 4:**
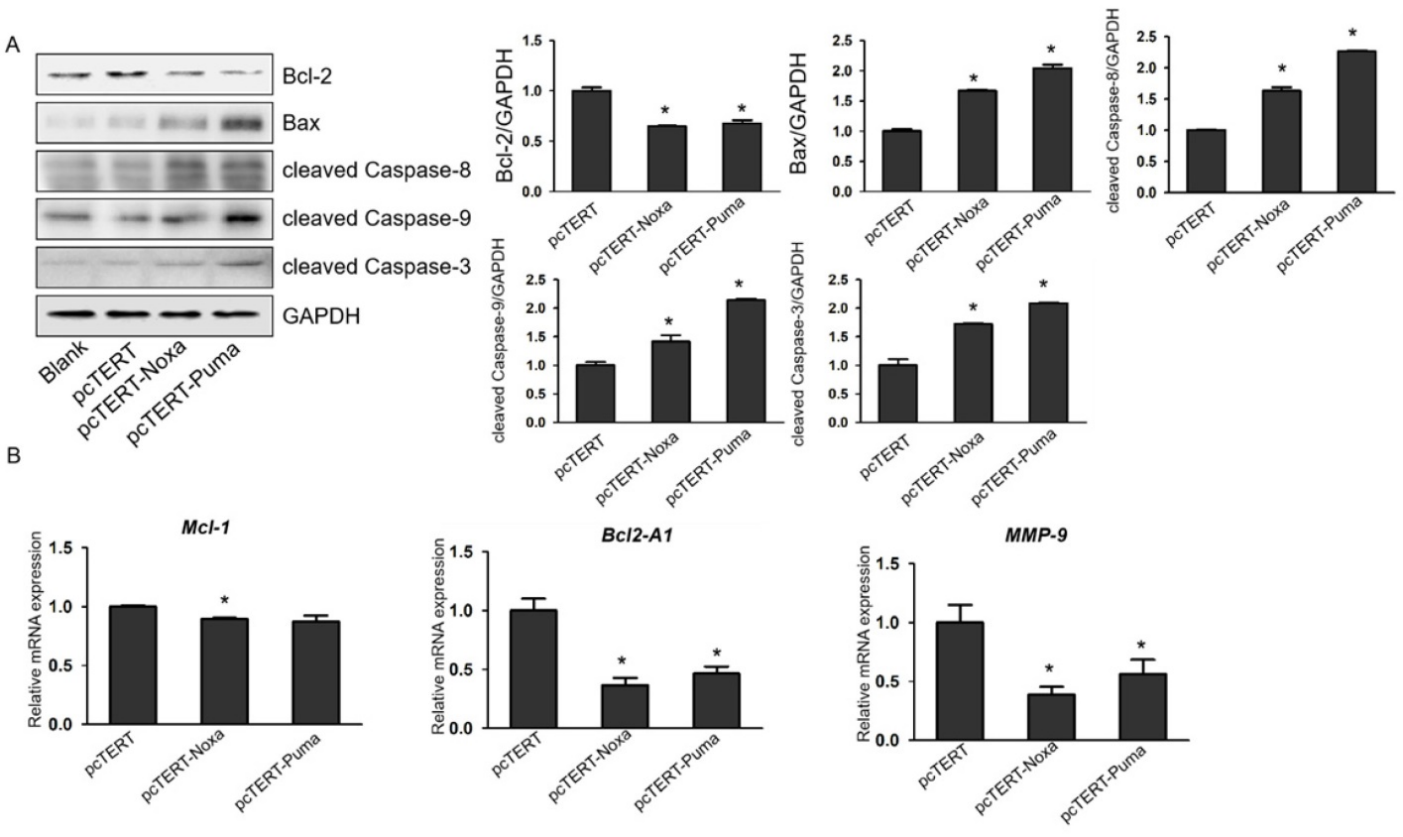
pcTERT-Noxa or pcTERT-Puma can trigger apoptosis in HepG2 cells by the mitochondrial and with death receptor pathways. (A) The expression levels of *Bcl-2*, *Bax*, *cleaved Caspase-3*, *cleaved Caspase-9* and *cleaved Caspase-8* were measured by western blot in HepG2 cells after transfection with pcTERT-Noxa or pcTERT-Puma for 72 h. (B) Relative mRNA expression of *Mcl-1*, *Bcl2-A1* and *MMP-9* was determined by Real-time PCR. The data has been represented as the mean ± SEM of three independent experiments. * *P*<0.05, pcTERT-Noxa *vs.* pcTERT, pcTERT-Puma *vs.* pcTERT**.** All the results of Western blot were repeated three times to obtain error bars and one representative blot of each group is shown. Each qPCR result was obtained from three independent experiments.

The migration and metastasis of tumors are greatly important indicators for better prognosis 31. To further study whether the pcTERT-Noxa or pcTERT-Puma can affect the migration of HepG2 cells, transwell assay was used. HepG2 cells stained in crystal violet of transwell chamber were dissolved in 30% acetate and OD570 was measured using a microplate reader. The OD570 value of pcTERT-Noxa (0.281±0.019) or pcTERT-Puma (0.250±0.009) treatment cells were found to be reduced significantly as compared with pcTERT control group (0.332±0.017) (Fig 3A). Moreover, the wound healing results have demonstrated that pcTERT-Noxa (26.30±1.29) %, (45.96±1.06) %, (60.82±0.61) % or pcTERT-Puma (19.80±2.68) %, (42.16±1.10) %, (57.04±1.50) % groups showed a significantly decrease in migration ability against pcTERT control group (34.36±1.58) %, (56.74±0.85) %, (70.51±0.65) % of HepG2 cells after 24, 48 and 72 h post-transfection (Fig 3B). Consistently, matrigel invasion experiment by using transwell assay indicated the ability of tumor cells to undergo invasion, which represented that the percentage of stained HepG2 cells that had significantly penetrated into the transwells were substantially reduced in pcTERT-Noxa (0.162±0.003) or pcTERT-Puma (0.160±0.003) groups, as compared with pcTERT (0.240±0.012) (Fig 3C). *MMP-9* has been reported to play a vital role in the process of cellular migration and invasion 32. It was also found that the expression of MMP-9 was obviously reduced in HepG2 cells transfected with pcTERT-Puma (38.93±6.59) % or pcTERT-Noxa (56.41±12.04) % as compared to pcTERT group (Fig 4B). Overall, these results suggested that that pcTERT-Noxa or pcTERT-Puma can significantly inhibit HepG2 cells migration and invasiveness.

### Noxa or Puma can lead to mitochondrial and death receptor pathways mediated apoptosis in HepG2 cells

It has been reported that overexpression of *Noxa* or *Puma* can promote apoptosis of HepG2 cells, but the underlying mechanisms remain poorly elucidated. *Caspase-3* plays a vital role in apoptotic process as *caspase-9*, upstream molecules such as *Bcl-2* and *Bax* were found to be involved in mitochondrial pathway and *caspase-8* was involved in death receptor pathway. Therefore, in our further studies, the protein levels of mitochondrial upstream mediators *Bcl-2*, *Bax* and downstream effectors *cleaved Caspase-9* and *cleaved Caspase-8* as well as *cleaved Caspase-3* were assessed. As shown in Fig. 4A, the expression of *Bax*, *cleaved Caspase-8*, *cleaved Caspase-9* and *cleaved Caspase-3* were substantially elevated in HepG2 cells transfected with pcTERT-Noxa or pcTERT-Puma in comparison with that in the pcTERT. Conversely, the level of *Bcl-2* was markedly reduced in the pcTERT-Noxa or pcTERT-Puma group as compared to pcTERT group, which was in accordance with the results that mRNA expression of *Bcl-2A1* was also significantly down-regulated in *Puma* (46.41±6.15) % or *Noxa* (36.50±6.16) % transfected groups (Fig 4B). In addition, the level of another *Bcl-2* family member *Mcl-1* was also found to be significantly reduced in pcTERT-Noxa (89.58±0.79) % group but not in pcTERT-Puma group (Fig 4B), which may be due to relatively higher within the group error of pcTERT-Puma (87.38±5.04) %. In summary, an overexpression of *Noxa* or *Puma* may lead to substantial apoptosis of HepG2 cells via mitochondrial and death receptor pathways, however the mechanisms regulating the apoptosis require additional investigations.

### Potential anti-tumor effect of the pcTERT-Noxa or pcTERT-Puma in H22-bearing mice

**Figure 5:**
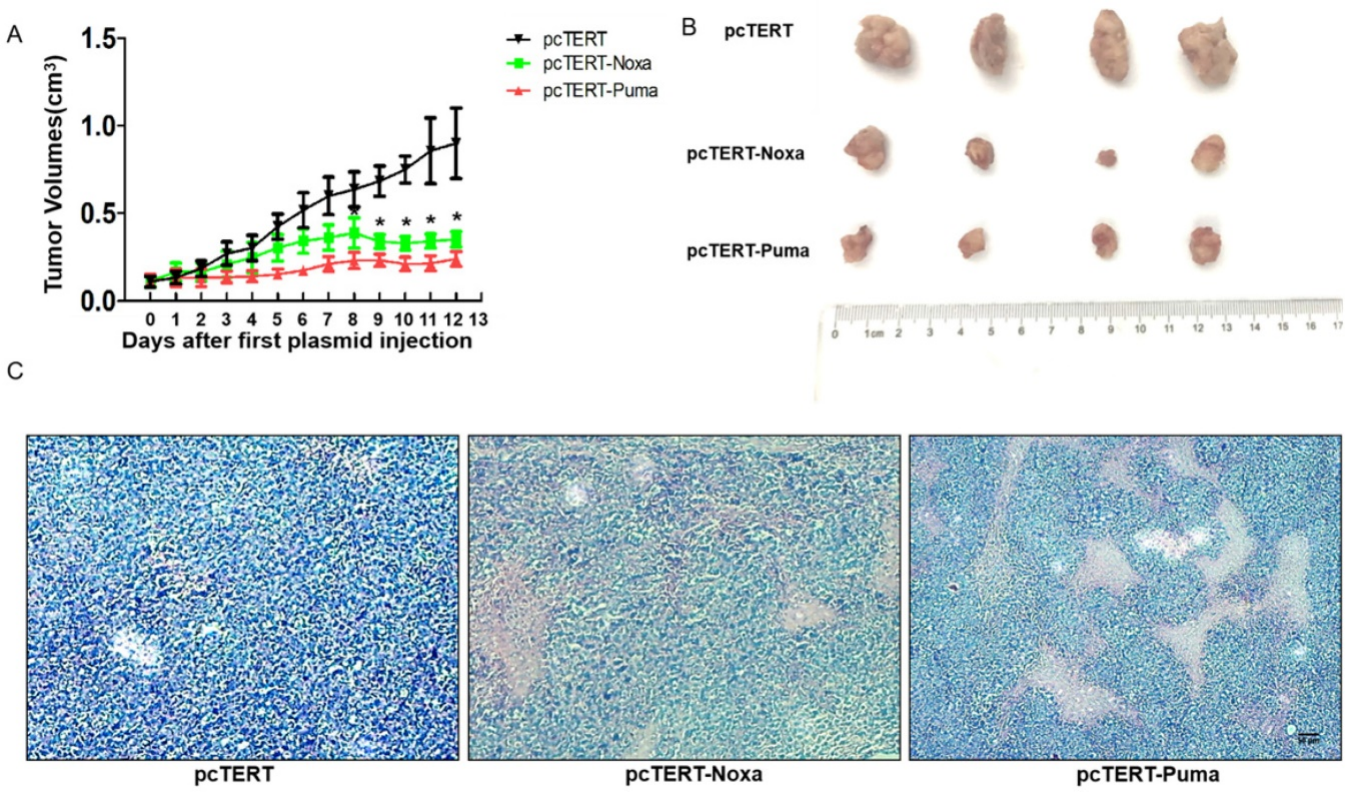
The anti-tumor effect of pcTERT-Noxa or pcTERT-Puma in H22-bearing mice (A) Tumor growth curves *in vivo* were reflected by changes in the tumor volumes post intratumoral injection of pcTERT, pcTERT-Noxa or pcTERT-Puma. Each group was performed with seven mice (B) The photographs of tumors were taken for each group, which were peeled off 3 days after the final plasmid injection. (C) Representative microscopic images of the H&E staining of H22-tumor cells after injections with plasmid pcTERT, pcTERT-Noxa or pcTERT-Puma in BALB/C mice (×40). * *P*<0.05, pcTERT-Noxa vs. pcTERT, pcTERT-Puma *vs.* pcTERT. The HE staining photos were collected from the tumor of each mouse and one representative picture of each group has been shown.

During *in vivo* study, the tumor growth was monitored everyday by calculating the tumor volume as described in “Method”. After the tumor mass became palpable (approximately 0.11 cm^3^), the mice were randomly divided into three different groups and administered with pcTERT, pcTERT-Noxa or pcTERT-Puma intratumoral injections. As shown in Fig 5A, the tumor volume displayed a rapid growth in a time-dependent manner in pcTERT group.

Nevertheless, in the pcTERT-Noxa or pcTERT-Puma groups, an inhibition in the tumor growth was obvious from the day 8 to the day 12 post the first injection compared with pcTERT controls. After receiving the last plasmid injection for 3 days, all the mice were sacrificed and tumors were taken as shown in Fig 5B, and the H&E staining was conducted (Fig 5C). The pathological changes as determined by H&E staining showed large areas of tumor cell death in pcTERT-Noxa or pcTERT-Puma groups as compared to pcTERT controls. Thus, the results illustrated that pcTERT-Noxa or pcTERT-Puma could significantly inhibit the tumor growth and finally lead to apoptosis of the tumor cells *in vivo*.

### pcTERT-Noxa or pcTERT-Puma can trigger apoptosis via enhancing T cell mediated-immunological response in mice

**Figure 6:**
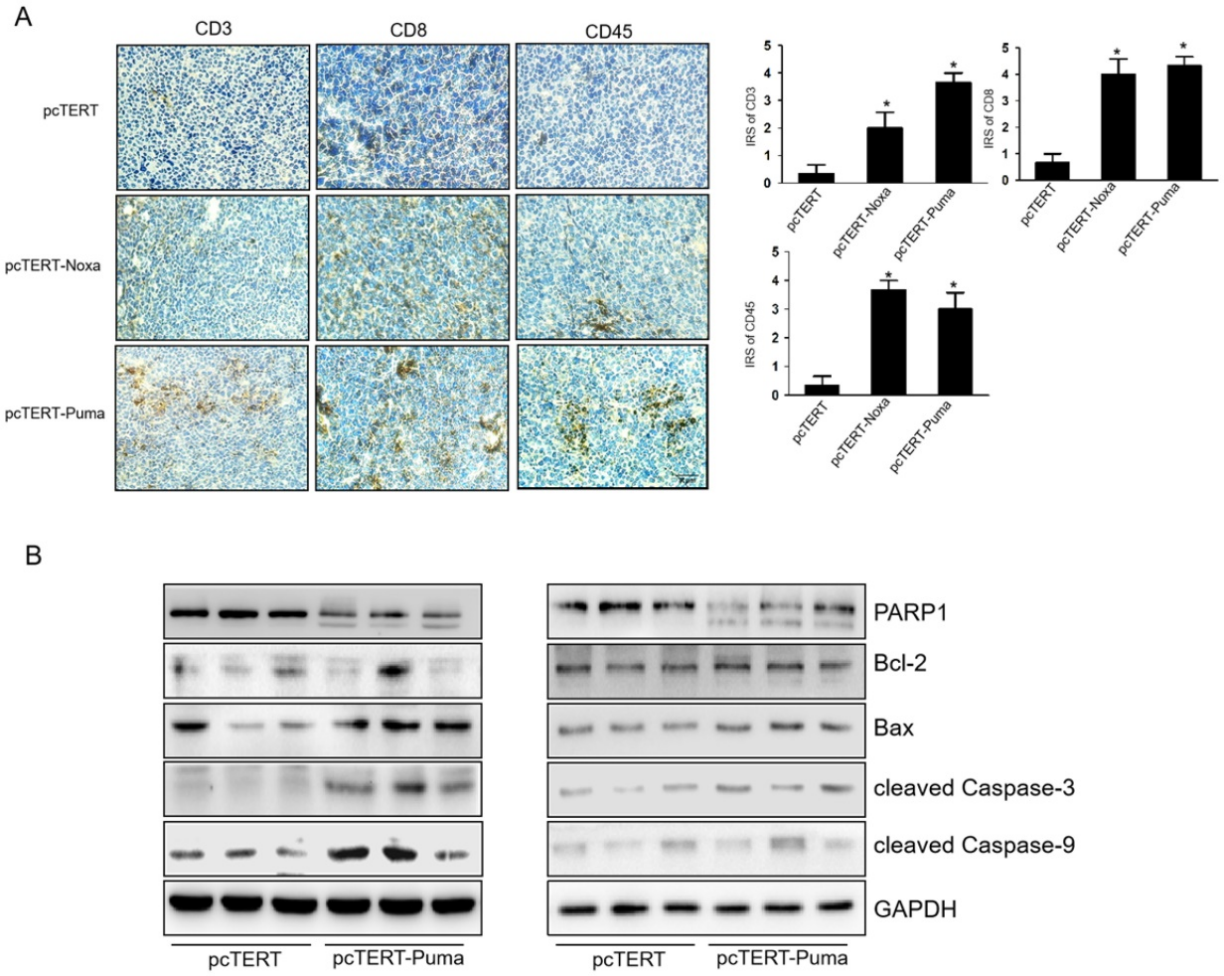
T-cell mediated anti-tumor effects of pcTERT-Noxa or pcTERT-Puma *in vivo* (A) *CD3^+^*, *CD8^+^* and *CD45^+^* T cells around the tumors upon injection of pcTERT, pcTERT-Puma or pcTERT-Noxa plasmids were determined by IHC (×100) (Left) and the immunoreactivity scores (IRS) were calculated by adding the immunostaining percentages and immunostaining intensity scores together (Right). (B) The expression of apoptosis related proteins regulating the mitochondrial pathway (*Bax*, *Bcl-2*, *PARP1*, *cleaved Caspase-9* and *cleaved Caspase-3*) in the tumor tissues was determined by western blot. * *P*<0.05, pcTERT-Noxa vs. pcTERT, pcTERT-Puma *vs.* pcTERT. Each experiment was performed with three mice. The IHC staining photos were collected from the tumor of each mouse and one representative picture of each group has been shown.

We next investigated the infiltrations of *CD3^+^*, *CD8^+^*,* CD45^+^* T-cell in the tumor after injections. IHC analysis showed that the numbers of *CD3^+^*, *CD8^+^* and CD45^+^ T-cells significantly increased in pcTERT-Puma or pcTERT-Noxa group as compared to pcTERT control (Fig 6A). We also detected the expression of apoptotic proteins in the tumors after pcTERT, pcTERT-Noxa or pcTERT-Puma injection. As shown in Fig 6B, tumor tissues obtained from pcTERT-Noxa or pcTERT-Puma groups showed a substantial increase in the expression of *cleaved PARP1*, *cleaved Caspase-9*, *cleaved Caspase-3* and *Bax* in tumor, which was similar to the *in vitro* results as shown in Fig 4A. However, *Bcl-2* expression indicated that there was no obvious variation in pcTERT-Noxa group or pcTERT-Puma group as compared to pcTERT injection group. Thus, the above findings revealed that transfection with pcTERT-Noxa or pcTERT-Puma plasmids may eradicate tumor cells by mitochondrial dependent apoptotic pathway via promoting the substantial accumulation of T cells.

## Discussion

Primary liver cancer (PLC) is a malignant tumor with a high incidence and lethality all over the world. *P53* is one of the most well-studied tumor suppressor genes and can play a central role in the cancer development 33. Despite the vital role of *p53* during tumor suppression, the molecules which play key roles downstream of *p53* in tumor suppression have still not been identified 34.The members of the BH3 only family, *Noxa* and *Puma* can function as the important downstream target genes of *P53* 35. In recent years, extensive research has been carried on *Noxa* and *Puma* proteins, and it has been found that these two proteins play a vital role in regulating the process of apoptosis 36.

In this study, after linking the human *Noxa* or *Puma* genes to the telomerase reverse transcriptase hTERT promoter to construct plasmids of pcTERT-Noxa or pcTERT-Puma, these two recombinant plasmids were transiently transfected into HepG2 cells respectively. The morphological changes in HepG2 cells were observed by optical microscopy after the transfection of pcTERT-Noxa or pcTERT-Puma plasmids for 48h or 72h. The results showed that cell shrinkage, apoptotic vacuoles formation and the disruption of cellular membrane occurred in pcTERT-Noxa or pcTERT-Puma transfection groups in a time-dependent manner. Annexin V-FITC/PI double staining and Hochest33258 are the typical methods which have been used over the years 37. Therefore, a further analysis with Annexin V-FITC/PI double staining and Hochest33258 staining assays were designed and clearly illustrated that transfection with pcTERT-Noxa or pcTERT-Puma induced significant apoptosis in HepG2 cells as compared to the pcTERT controls. CCK-8 and colony formation assay also established that the substantial inhibition in proliferation was caused by pcTERT-Noxa or pcTERT-Puma plasmids in HepG2 cells specially, not in normal HL-7702 liver cells. These finds illustrated that *Noxa* or *Puma* can stimulate apoptosis and significantly inhibit the growth of HepG2 cells, which was consistent with the results in other cancer cells 38, 39.

Upon receiving an apoptosis signal, the complex of apoptotic related protein *Bax* and anti-apoptotic protein *Bcl-2* can tightly aggregate along the surface of mitochondrial membrane. This can effectively result in the decline in membrane potential and an increase in membrane permeability, thereby releasing *Cyt C* into the cytoplasm and binding with *Apaf-1* (apoptotic protease-activating factor-1) and finally leading to the formation of an apoptotic complex 40. The apoptosis complex can recruit *pro*-*caspase-9* and further activate the effector *caspase-3*, to initiate the caspase mediated cascade of events including *PARP1* mediated-DNA cleavage that can finally lead to cellular apoptosis 40. It has been reported that *Noxa* and *Puma* can be activated by *P53* triggered mitochondrial pathway of apoptosis in colon cells but the detailed mechanism(s) remain unclear 35. In the present study, after transfection with pcTERT-Noxa or pcTERT-Puma plasmids in HepG2 cells, the levels of upstream mitochondrial apoptosis regulator Bcl-2 and Bax were substantially altered, which was followed by an increase in expression of *cleaved Caspase-9*, *cleaved Caspase-3* and *cleaved PARP1* in HepG2 cells in the comparison with pcTERT control. Besides, the potential effects of pcTERT-Noxa and pcTERT-Puma *in vivo* were studied by intratumoral injection of these two plasmids into H22 tumor-bearing BALB/C mice. PcTERT-Noxa or pcTERT-Puma injection exhibited significant tumor growth suppression as compared with pcTERT after 8 days post plasmid injection, which was further validated by H&E staining. In recent years, the anti-tumor effects of T cells mediated tumor immunotherapy have gradually drawn great attention 41. A number of studies have shown that the tumor-infiltrating T cells can exhibit significant antitumor effect by binding to the specific antigens of the targeting cells 42. *CD3* and *CD8* are considered as the T-cell-specific marker 43 whereas *CD45* belongs to the common leukocyte antigen that can play a vital role in regulation of T-cell and B-cell antigen receptor signaling 44. We found that tumors injected with pcTERT-Noxa or pcTERT-Puma plasmids displayed an increased expression of *CD3*, *CD8* and *CD45* as compared to pcTERT control injection group. This clearly illustrated that the anti-tumoral effects of pcTERT-Noxa or pcTERT-Puma may be partly mediated by altering infiltration of *CD3+*, *CD8+*and *CD45+* T cells triggered by the increased expression of *Noxa* or *Puma* in the tumor tissues. Moreover, mitochondrial related proteins *Bax*, *cleaved Caspase-3*, *cleaved Caspase-9*, and *cleaved PARP1* levels were stimulated after pcTERT-Noxa or pcTERT-Puma injection in the tumor tissues *in vivo*, which was consistent with the *in vitro* results as described above and have also addressed some limitations associated with our previous study 26. The expression of *Bcl-2* was not altered after pcTERT-Noxa or pcTERT-Puma injection, which needs further investigation.

Tumor migration and invasion are the principal steps involved in tumor metastasis that can serve as key factors in patient prognosis and tumor recurrence 43. The MMP family members can regulate tumor invasion and migration, by causing an effective degradation of the basement membrane 45. Hence, the ability of migration and invasiveness of HepG2 cells was examined using the transwell chambers and cell scratch assay, and the expression of migration related genes was also examined after transfection with pcTERT-Noxa or pcTERT-Puma plasmids. The results showed that *Noxa* or *Puma* significantly reduced the ability of tumor cells to undergo migration and invasion as compared with the control plasmid. Consistent with these results, the level of *MMP-9* protein was also significantly decreased by pcTERT-Noxa or pcTERT-Puma, thereby indicating the significant inhibitory effects of pcTERT-Noxa or pcTERT-Puma transfection on the migration and invasion of HepG2 cells. Moreover, another previous study has indicated that *P53* was associated with tumor migration and invasion 46, but it was not clear whether the downstream molecules of *P53* were also involved in the process. Our findings have established new concrete mechanisms that can explain the role of *P53* in regulating the tumor migration and invasion.

The present study illustrated the potential anticancer actions of two novel recombinant pcTERT-Noxa and pcTERT-Puma plasmids, which contained the BH3 only family members* Noxa* or *Puma* harboured within the hTERT promoter. It was demonstrated that the pcTERT-Noxa or pcTERT-Puma plasmids induced apoptosis in HepG2 cells and H22-bearing mouse tumor tissues were mediated via the mitochondrial pathway, and these constructs did not affect the viability of the normal liver cells HL-7702. pcTERT-Noxa or pcTERT-Puma also inhibited the migration and invasion of the HepG2 cells, thereby suggesting that they may form the basis of novel therapeutic strategy against liver cancers.

## Figures and Tables

**Table 1:** Primers used for the quantitative real-time PCR**.**

Genes	Forward primers (5' to 3')	Reverse primer (5' to 3')
*Puma*	GGAGGGTCCTGTACAATCTC	GTGCAGGCACCTAATTGGG
Noxa	GAAGAAGGCGCGCAAGAAC	GGTTCCTGAGCAGAAGAGTTTG
*BAX*	GGCAACTTCAACTGGGGC	CCACCCTGGTCTTGGATCC
*Bcl-2*	AGGATTGTGGCCTTCTTTGA	TCAGGTACTCAGTCATCCAC
*Mcl-1*	GGCGCCAAGGACACAAAGCC	CCAACCCGTCGTAAGGTCTC
*Bcl2-A1*	CGTAGACACTGCCAGAACAC	TCCACATCCGGGGCAATTTG
*MMP-9*	ACCTGGGCAGATTCCAAACCT	CGGCAAGTCTTCCGAGTAGT
*GAPDH*	GCACCACCAACTGCTTAG	GCAGGGATGATGTTCTGG
